# Carvacrol: An *In Silico* Approach of a Candidate Drug on HER2, PI3K*α*, mTOR, hER-*α*, PR, and EGFR Receptors in the Breast Cancer

**DOI:** 10.1155/2020/8830665

**Published:** 2020-10-26

**Authors:** Oscar Herrera-Calderon, Andres F. Yepes-Pérez, Jorge Quintero-Saumeth, Juan Pedro Rojas-Armas, Miriam Palomino-Pacheco, José Manuel Ortiz-Sánchez, Edwin César Cieza-Macedo, Jorge Luis Arroyo-Acevedo, Linder Figueroa-Salvador, Gilmar Peña-Rojas, Vidalina Andía-Ayme

**Affiliations:** ^1^Academic Department of Pharmacology, Bromatology and Toxicology, Faculty of Pharmacy and Biochemistry, Universidad Nacional Mayor de San Marcos, Jr Puno 1002, Lima 15001, Peru; ^2^Chemistry of Colombian Plants, Institute of Chemistry, Faculty of Exact and Natural Sciences, University of Antioquia-UdeA, Calle 70 No. 52-21 A.A, Medellin 1226, Colombia; ^3^University of Pamplona, Faculty of Basic Sciences, Colombia, Km 1 Vía Bucaramanga Ciudad Universitaria, Pamplona, Colombia; ^4^Laboratory of Pharmacology, Faculty of Medicine, Universidad Nacional Mayor de San Marcos, Av. Miguel Grau 755, Lima 15001, Peru; ^5^Laboratory of Biochemistry, Faculty of Medicine, Universidad Nacional Mayor de San Marcos, Av. Miguel Grau 755, Lima 15001, Peru; ^6^Laboratory of Physiology, Faculty of Medicine, Universidad Nacional Mayor de San Marcos, Av. Miguel Grau 755, Lima 15001, Peru; ^7^School of Medicine, Faculty of Health Sciences, Universidad Peruana de Ciencias Aplicadas, Prolongación Primavera 2390, Lima 15023, Peru; ^8^Laboratory of Cellular and Molecular Biology, Biological Sciences Faculty, Universidad Nacional de San Cristóbal de Huamanga, Portal Independencia 57, Ayacucho 05003, Peru; ^9^Laboratory of Food Microbiology, Biological Sciences Faculty, Universidad Nacional de San Cristóbal de Huamanga, Portal Independencia 57, Ayacucho 05003, Peru

## Abstract

Carvacrol is a phenol monoterpene found in aromatic plants specially in Lamiaceae family, which has been evaluated in an experimental model of breast cancer. However, any proposed mechanism based on its antitumor effect has not been reported. In our previous study, carvacrol showed a protective effect on 7,12-dimethylbenz[*α*]anthracene- (DMBA-) induced breast cancer in female rats. The main objective in this research was to evaluate by using *in silico* study the carvacrol on HER2, PI3K*α*, mTOR, hER-*α*, PR, and EGFR receptors involved in breast cancer progression by docking analysis, molecular dynamic, and drug-likeness evaluation. A multilevel computational study to evaluate the antitumor potential of carvacrol focusing on the main targets involved in the breast cancer was carried out. The *in silico* study starts with protein-ligand docking of carvacrol followed by ligand pathway calculations, molecular dynamic simulations, and molecular mechanics energies combined with the Poisson–Boltzmann (MM/PBSA) calculation of the free energy of binding for carvacrol. As result, the *in silico* study led to the identification of carvacrol with strong binding affinity on mTOR receptor. Additionally, *in silico* drug-likeness index for carvacrol showed a good predicted therapeutic profile of druggability. Our findings suggest that mTOR signaling pathway could be responsible for its preventive effect in the breast cancer.

## 1. Introduction

Carvacrol (2-methyl-5-(1-methylethyl)-phenol) is a phenol monoterpene and represents the major phytochemical in the essential oil of aromatic vegetable species belonging to the family Lamiaceae [1]. Some species with high content of carvacrol are *Origanum vulgare* (16.2%–81.92%), *Origanum acutidens* (76.21%) [2], *Thymus vulgaris* (43.8%)*, Thymus kotschyanus* (24.4%), *Thymus kotschyanus* (24.4%), *Thymus capitatus* (>80%) *Thymus caramanicus*, *Thymus fallax* (from 50% to 70%), and *Thymus algeriensis* (7.8%) [3]. In regard to *Thymus vulgaris*, which is a species very cosmopolite in the world, the content of carvacrol can vary; this is due to the different chemotypes based on its volatile chemical composition. In Europe, *Thymus vulgaris* revealed at least 20 different chemotypes types, which carvacrol may range between 2% and 8% [4]. Otherwise, the essential oil of *Origanum vulgare* known as oregano also presents high variability of carvacrol linked to stage of harvest, ecological and climatic parameters ranging in countries such as Saudi Arabia (70.2%), Brazil (4.7%) [5], Italy (21.89%) [6], and Kashmir in the Himalaya region (52.3%–84.54%) [7].

Investigations related to its pharmacological activity have been tested by using *in vitro* assays overall in cell cultures such as anti-inflammatory, anticancer, antimicrobial, antifungal, antioxidant, antiapoptotic, antiproliferative, anti-invasion, and cytotoxic activities in order to establish any involved molecular mechanisms of carvacrol [8, 9]. Many molecules from nature sources are studied to determine if there could be a novel candidate as an antitumor potential drug yearly. When a molecule is isolated and characterized chemically, the *in silico* studies are the first step in the basic research that leads to the following stage of evaluation as the *in vitro* and *in vivo* studies. The *in silico* tests could exert the main mechanism on a biological target as well as its pharmacokinetic profile.

Nowadays, some citable works corresponding with the antitumor activity of carvacrol *in vitro* and *in vivo* have been reported. Although carvacrol is an old chemical component isolated from the essential oils of aromatic plants, recently, in the last decade, mechanisms involved in the tumorigenesis of certain types of cancer as prostate, lung, breast, gastric and colon have been evaluated only *in vitro* and recently employing experimental animals in breast and colon cancer. Even though the toxicity of carvacrol was evaluated in animals, this data is limited. It has been reported that the median lethal dose of carvacrol in rats is 810 mg/kg of body weight by oral gavage. Additionally, in an animal model of cancer induction, the maximum doses tested were 200 mg/kg of body weight and side effects linked to body weight loss and death were not evidenced [10].

The autophagy and adipogenic differentiation of about 30%–40% produced by carvacrol could be the cause to stop the cancer progression. It has been demonstrated that inhibition of mTOR by MEK signaling and LC3B-II expression promotes autophagy induction in an *in vitro* model of human cervical cancer. Additionally, carvacrol nanoemulsion suppresses autophagy through enhancing the decreased conversion of autophagy-related genes LC‐3 I to II, downregulating ATG5 (autophagy related 5) and ATG7 (autophagy related 7), and upregulating the protein P62. On the other hand, carvacrol downregulated the PI3K/AKT signaling on MCF-7 cells (human breast adenocarcinoma cells) [11]. Even though carvacrol has shown antiproliferative effects on a human metastatic breast cancer cell line (MDA-MB 231) and human non-small-cell lung cancer cell line (A-549), its mechanism is associated with biochemical changes in the mitochondria such as depolarization of the membrane potential, release of cytochrome c, and activation of caspase producing apoptosis [12, 13].

Additionally, carvacrol has demonstrated cytotoxicity against human cervical cancer cells HeLa [14], as well as antiproliferative and apoptotic on human liver cancer cells HepG-2 [15], cytotoxicity in AGS human gastric adenocarcinoma cells [15], liver cancer in rats [16], apoptosis in human oral squamous cell carcinoma (OSCC), and antiproliferative in N2a neuroblastoma cells; in PC-3 prostate, cancer cells reduced the IL-6 protein levels, pSTAT3, pERK1/2, and pAKT signaling proteins. Furthermore, it exerted an antiproliferative effect on DU-145 prostate cancer cells by inhibiting TRPM7 channels and suppression of PI3K/Akt and MAPK signaling pathways [17, 18].

We proposed an *in silico* analysis and molecular docking studies on the main targets involved in the progression of the mammary tumors such as phosphatidylinositol-3-kinase-*α* wild type (PI3K*α*), human estrogen receptor-*α* (hER-*α*), progesterone receptor (PR), human epidermal growth factor receptor-2 (HER2), the mammalian target of rapamycin (mTOR), and epidermal growth factor receptor (EGFR), as well as molecular dynamic simulation and molecular mechanics energies combined with the Poisson–Boltzmann (MM/PBSA) studies, as well as evaluating its drug-likeness properties *in silico* in order to demonstrate druggability.

## 2. Results and Discussion

### 2.1. Multitarget Molecular Docking Investigation

Cancer in mammals involved multiple biological targets responsible for tumor cell proliferation and survival. Indeed, these receptors are included within key cellular signaling pathways which commonly appear to be genetically amplified in different tumors like breast cancer, including PI3K/Akt/mTOR signaling, HER2/EGFR-AKT system, and the classical hormone receptor‐positive (ER+) pathway, which are implicated in the tumor self-renew, survival, and proliferation in breast cancer [19–22]. Targeting a single or multiple signaling pathways is considered nowadays a promising strategy for anticancer chemotherapy. Despite drugs available to treat breast cancer (i.e., lapatinib, alpelisib, rapamycin, tamoxifen, gefitinib, and the repurposing drug ulipristal acetate) have beneficial profiles, the severe and life-threatening side effects many of them have are notable. It has led to accelerated development of novel chemotherapy alternatives, which computational approaches have been strongly employed for understanding drug-protein interactions, as well as mechanistic study when the potential drugs are placed within target-active site and binding affinity is calculated.

In this context, marked biological effects both *in vitro* and *in vivo* for carvacrol against breast cancer cells described here inspired further studies. For the current investigation, we hypothesized that carvacrol at least might target one of the signaling pathways, providing a plausible explanation from the observed experimental results. Thus, in order to afford an insight at a possible molecular-level mechanism for this compound, we performed docking investigations against the most valuable therapeutic targets for breast cancer therapy, such as phosphatidylinositol-3-kinase-*α* wild type (PI3K*α*), human estrogen receptor-*α* (hER-*α*), progesterone receptor (PR), human epidermal growth factor receptor-2 (HER2), the mammalian target of rapamycin (mTOR), and epidermal growth factor receptor (EGFR), which as previously mentioned play a fundamental role in breast cancer growth, invasiveness, and metastasis. Rigid receptor docking was performed in order to provide structural insights into the binding mode of carvacrol into every refined cancer signaling proteins.

To accomplish this goal, we calculated the binding energy scoring function of carvacrol docked against the *X*-ray crystallographic structures of these key proteins associated with breast cancer as follows: HER2 (PDB ID: 3PP0), PI3K*α* wild type (PDB ID: 4JPS), mTOR (PDB ID: 4DRI), hER-*α* (PDB ID: 3ERT), PR (PDB ID: 4OAR), and EGFR (PDB ID: 3POZ). In addition, we have screened six FDA-approved drugs for breast cancer, as well as strong inhibitors and its binding affinities were also determined to ensure certain amount of confidence regarding the Autodock scoring function of this project. The binding free energies produced by docking action of carvacrol and the known inhibitors on each catalytic site of the selected key protein targets are tabulated in Table 1.

As shown in Table 1, docking results showed that the best binding interaction was founded when carvacrol was docked with mTOR. In fact, a critical exploration of the selected active sites revealed that the binding pockets for the majority of these targets are too large to accommodate well carvacrol and achieve protein docking interactions, but not for the mTOR protein complex. Carvacrol had a docking score value of −7.5 kcal·mol against mTOR, which notably appears to be close to those founded for the rapamycin in this work (−8.6 kcal·mol) and previous works (−8.4 kcal·mol).

#### 2.1.1. Docking Profile inside mTor Active Domain for Carvacrol: A Single-Target Approach

Due to interestingly binding energy founded for carvacrol against X-ray crystallographic structure of mTOR, an effective multilevel computational study based on single-objective involved docking followed by MD simulation and MM/PBSA free energy calculations was performed aiming to explore the potential of carvacrol to inhibit the mTOR function. The mammalian or mechanistic target of rapamycin (mTOR) is a complex metabolic pathway responsible for activating cellular sensor to nutrients, cell growth, and proliferation in breast cancer; its inhibition is a promising therapeutic opportunity for breast cancer therapy [23, 24]. The architecture of mTOR complexes has been solved in detail by März et al. and cocrystallized with the cyclic macrolide rapamycin. The structure explains how rapamycin is capable of inhibiting mTOR function by binding to small protein termed as FKBP12, and how resulting complex then interacts with the FRB-mTor domain. This interaction disrupts the association of mTOR with the catalytic domain of mTORC1 and may block nutrient signaling and cell growth in breast cancer [25]. Thus, the most important active pocket into the mTOR pathway comprise the interface between FKBP12 and the FRB-mTor domain (namely, as rapamycin-binding pocket) and enclosing key binding interactions which play a crucial role in the catalytic activity of the kinase, including twelve contacts into FRB-mTor domain with His2028, Glu2033, Tyr2104, Leu2097, Gln2099, Trp2101, Tyr2038, Arg2036, Phe2108, Leu2031, Tyr2105, and Phe2039 and twenty residues from FKBP12, such as Arg73, Tyr113, Ile87, Asp68, Gln85, Gly84, Val78, Phe79, Leu128, Gly59, Lys121, Phe130, Lys88, Ser118, Ile122, Phe67, Tyr57, Trp90, Val86, and Phe77. Thus, docking investigations were carried out using final dimensions of the grid box of 32 Å × 32 Å × 32 Å and set on *X* = 34.343, *Y* = 48.363, and *Z* = 38.034, centering around key residues.

**Figure 1:**
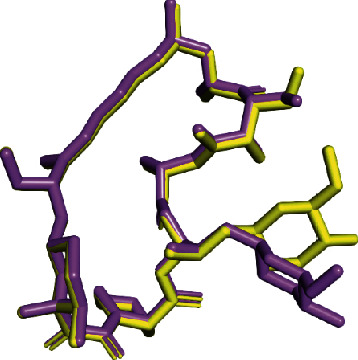
Self-docking validation. Alignment of the best-docked pose of rapamycin (in violet) and the crystallographic binding mode (in yellow).

In order to accomplish high throughput, AutoDock Vina protocol inside mTOR binding pocket was firstly validated through self-docking. We performed a comparison of the crystallographic binding mode of the rapamycin deposited in PDB by März et al. and the lowest energy docking pose. To carry out this validation, root-mean-square deviation (RMSD) value was calculated to correlate the differences between the atomic distances. As shown in Figure 1, the docked conformation predicted for rapamycin (in violet) is spatially close to the crystallographic structure pose (in yellow) with an optimal RMSD value of 1.44 Å. In addition, as shown in Table 1, the best binding energy calculated for rapamycin (−8.6 kcal·mol) was in good agreement with the literature data (−8.4 kcal·mol) [26]. These findings indicate a high-level of the feasibility in our protein-ligand docking procedure, which was able to reproduce the binding pose of the cocrystallized ligand deposited in the PDB ID: 4DRI.

**Figure 2:**
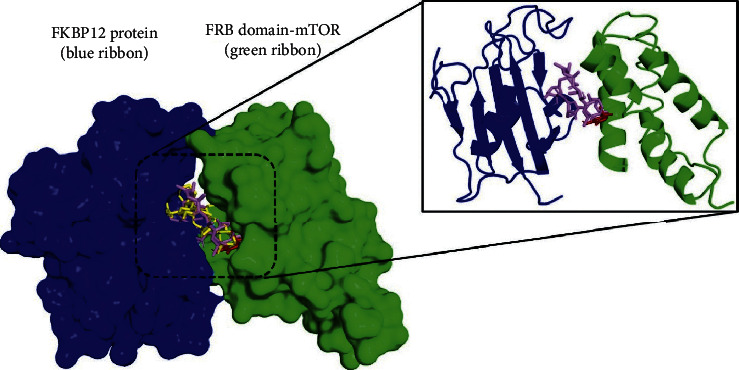
Superposition of the best pose-docked of carvacrol and rapamycin at the interface cavity of FKBP12 and mTOR-FRB domain. Carvacrol (in red), cocrystallized pose for rapamycin (in yellow), and best-docked pose for rapamycin (in violet).

After the docking protocol is validated, an exhaustive search in the binding pocket was carried out in order to establish key binding site points when carvacrol was docked into rapamycin binding Site of the mTOR catalytic domain. To this purpose, the best binding conformation for carvacrol and rapamycin was analyzed to make a valid comparison. A simple visual inspection to the superimposition of the docked inhibitors and carvacrol revealed that the carvacrol had a docked structure that fit well within the rapamycin-binding site with a low binding energy of −7.5 kcal·mol (Figure 2).

As rapamycin, carvacrol was also able to bind to mTOR with at least eleven essential amino acids for the catalytic activity of mTOR as follows: His2028, Glu2033, Tyr2104, Phe2108, Leu2031, Tyr2105, and Trp2101 at the mTOR-FRB domain, as well as Val78, Phe79 Val86, and Phe77 with the FKBP12 protein (Table 2). This particular result supports our proposal: carvacrol might block mTOR function with similar binding affinity to rapamycin preventing the cell growth and proliferation.

**Figure 3:**
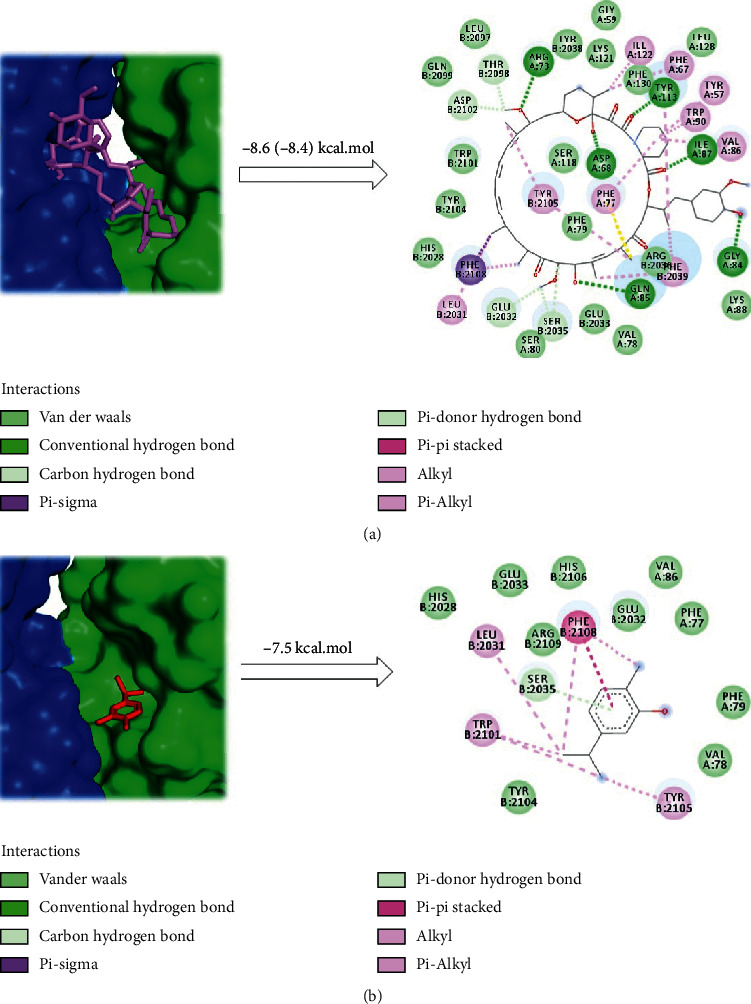
2D ligand-protein interaction plots with the 3D-crystal structure of mTOR: (a) rapamycin and (b) carvacrol.

Furthermore, this preliminary finding was also supported by an inspection of the 2D protein-ligand interaction plot after the docking protocol for carvacrol, which revealed similar key interactions in comparison with rapamycin (Figure 3(a)). Thus, carvacrol displays the occurrence of seven *σ/π*-*π* interactions into the mTOR-FRB domain with key Phe2108, Leu2031, Tyr2105, Trp2101 residues, as well as hydrophobic interactions with three residues postulated to bring about the catalytic function of mTOR (His2028, Glu2033, and Tyr2104). Furthermore, van der Waals contacts were formed between carvacrol with four residues of FKBP12 protein, such as Val78, Phe79 Val86, and Phe77. Finally, we also observed further interactions of carvacrol with mTOR, including five contacts with mTOR–FRB domain that have not been reported yet for current mTOR inhibitors as follows: one *π*-H-bond interaction with Ser2035 and four hydrophobic interactions surrounded by side chains of Arg2106, Glu2032, and His2106 (Figure 3(b)). Both crucial interactions and those additional binding interactions might contribute to increasing mTOR affinity; hence carvacrol can tightly bind to the mTOR and potently could inhibit its activity, suggesting that this small molecule may become a better drug prototype against breast cancer by targeting mTOR pathway.

#### 2.1.2. Docking-Based Molecular Dynamics Simulation

Molecular docking followed by MD simulation and MM/PBSA studies is a multilevel computational strategy to facilitate the process of drug designing against cancer. In fact, combining these computational protocols may conduce to develop safe and effective therapeutic options in response to the breast cancer [27]. Thus, in order to verify the docking computational solution obtained for carvacrol against the potential target mTOR protein, the best-docked pose of carvacrol into rapamycin-binding site was subjected to MD dynamic studies at 50 ns to explore the stability for ligand-protein complex, followed by MM/PBSA studies aiming to calculate the binding free energy of mTOR-carvacrol complex. Atom positional RMSD values in general equilibrate quickly during MD simulation, whereas an average RMSD value of 2.52 ± 0.02 Å was obtained and fall within the optimal range around 2 Å [28, 29]. This interesting finding suggest that the mTor-carvacrol complex predicted by molecular docking tends to reach dynamic stability at least in the time of 50 ns.

**Figure 4:**
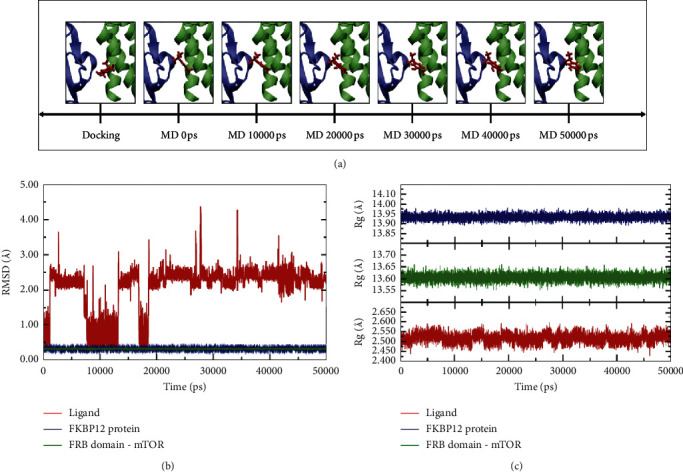
(a) Graphical snapshots at different periods during the MD simulation. (b) RMSD of the backbone of the carvacrol into the mTOR binding domain (in red). (c) Radius of gyration (Rg) plot for carvacrol into the binding pocket (in red) and every protein without ligand (green and blue).

As illustrated in Figures 4(a) and 4(b), after 50 ns MD simulation the starting carvacrol docking pose exhibited two strong fluctuations in RMSD at around 10 and 18 ns, but notably achieved equilibrium beyond 20 ns within cavity-ligand binding. RMSD fluctuations primarily can be attributed as binding pocket large and elongated promoting large accommodations of the aromatic ring into the active site. Within the limitations of this study, this preliminary conclusion is based on the observations provided by Figures S1 and S2 in the supplementary material.

On the other side, the radius of gyration (Rg) represents the compactness of the protein structure and conformational stability of the whole systems (i.e., protein-ligand complexes). We performed Rg analysis to observe the conformational alterations and dynamic stability of the carvacrol into the mTOR-FRB domain. The predicted values of Rg for carvacrol (2.05 ± 0.64 Å) are listed in Figure 4(c). Rg value confirms the stabilization and suggests there was no significant change in the residual backbone and folding of the mTor protein after the binding process with carvacrol. This finding suggests not only that ligand-protein interactions remain intact during the simulation period, but also the protein-ligand structure is not disturbed over the entire trajectory (50 ns). The above-mentioned statement was also supported by 2D-binding interactions maps and 3D representation of carvacrol in the mTOR catalytic pocket (see supporting information in Figures S1–S3). Notably, trajectory snapshots extracted along MD simulation every 10 ns revealed that four of those key interactions established by the docking studies, which are essential to the mTOR function, were maintained stable during the simulation period, such as Leu2031, Trp2101, Ser2035, and Tyr2105. In addition, further interaction was evidenced with residue Phe2039 (FRB domain), which has been demonstrated to play an important role in the mTOR function [19, 30, 31] and also as part of those interactions with the rapamycin inhibitor (Table 2). These crucial interactions of mTOR with carvacrol are the probable reason for its marked antiproliferative activity.

Furthermore, 3D representation of carvacrol into mTOR binding pocket was used to make a comparison between the top-scoring binding pose and the equilibrated conformation after 50 ns MD simulations; hence we plotted the superposition of the docked complex 3D-structures before and after MD simulation into de catalytic domain (see supporting information in Figures S2 and S3). In general, there are no dramatic differences between the structures extracted after 50 ns MD simulation and the best docking pose of carvacrol.  Figure S3 showed that the aromatic ring in the small molecule is slightly shifted; indeed this slight rotational motion favored its contact with the key residue Phe2039, which as above-mentioned apparently plays a critical role in mTOR activation.

The obtained MD simulation results suggest (1) the initial docking conformation of the binding pocket and carvacrol were stable during the 50 ns MD simulations, (2) carvacrol does not leave the binding pocket while running MD simulation, and (3) crucial binding interactions initially shown by the docking results were maintained throughout the MD simulation; indeed we could find strong evidence that carvacrol may be able to interact with the key Phe2039 residue located within receptor binding domain of FRB, which becomes clearly visible when MD simulations were carried out. These findings not only suggested the rationality and validity of the active conformations obtained using AutoDock, but also proposed that carvacrol could act as rapamycin-like inhibitor of mTOR complex, which is highly implicated in the protein synthesis, cell growth, and cell proliferation in human breast cancer tissues.

#### 2.1.3. MM/PBSA Binding Free Energy Calculations

Finally, molecular mechanics combined with Poisson−Boltzmann and surface area (MM/PBSA) calculations were carried out in order to estimate the different contributions to the binding free energy during mTOR-carvacrol complex formation, which were obtained from a standard single-trajectory MMPBSA protocol. Use of a postdocking procedure based on MMPBSA approach plays an increasingly important role in understanding many subjects in molecular modeling studies focus on clinical applications, leading to development of anticancer compounds [32, 33]. To address this, MM/PBSA calculations were performed using the g_mmpbsa package [34] (from the last 40 ns of trajectories from the production stage) to obtain free-energy contributions to the mTOR-carvacrol complex stabilization, which are summarized in Table 3.

From the results of MM/PBSA studies (Table 3), a molecular understanding of the binding interaction between carvacrol to the potential target (mTOR) by estimating different components of interaction energy that contributes to this binding was performed. Carvacrol possesses high nonbonded interaction energy with mTOR indicating its strong binding affinity (ΔG_bind_ value of −18.03 ± 1.57 kcal·mol^−1^). Moreover, van der Waals contacts have a greater energy contribution (ΔG_vdw_ = −19.28 ± 1.46 kcal·mol^−1^) favoring the carvacrol bind to mTOR, while solvent accessibility (ΔG_SASA_ = −2.21 ± 0.14 kcal·mol^−1^) and electrostatic interactions (ΔG_Electr_ = −0.38 ± 0.70 kcal·mol^−1^) only slightly contributed to total free binding energy. These findings revealed that those favorable ligand binding contributions could play a crucial role in the inhibition of mTOR with carvacrol inside the rapamycin binding site. Besides, unfavorable polar contributions were seen for carvacrol binding by the positive value obtained after MM/PBSA runs (3.85 ± 1.46 kcal·mol^−1^).

In addition, a per residue binding free energy decomposition using MM/PBSA was carried out in order to estimate individual energy contributions of each residue to the total binding free energy. As listed in Figure S4 in the Supplementary Information, per residue binding energy decompositions by using Amber MMPBSA showed that the key surrounding residues on complex carvacrol-mTOR resulted in being consistent with those found to be important to mTOR function and were in good agreement with close contacts resulting from MD simulations; among them, we can highlight Asp68, Glu75, Phe77, Gln85, and Asp91 (from the FKBP12 protein) and Leu2031, Ser2035, Phe2039, Trp2101, Asp2102, Tyr2104, Tyr2105, and Phe2108, from the FRB domain. From the calculations, we also observed that Lys52, Arg73, Lys83, and Lys121 (from the FKBP12 protein) and Arg2042, Arg2109, and Arg2110 (from the FRB domain) represent energetically unfavorable contacts during the ligand-binding event.

From the data collected by MM/PBSA calculations, it is suggested that van der Waals forces together with electrostatic interactions and the solvation free energy contributes to the mTOR-carvacrol complex stability. Importantly, not only carvacrol may bind to mTOR primarily through hydrophobic interactions mostly from the mTOR-FRB domain, but also may equally bind favorably and strongly to the rapamycin-binding site compared to rapamycin.

#### 2.1.4. Carvacrol Drug-Likeness Evaluation

Currently, early prediction of drug-likeness filters provides a useful guideline for further optimization of small molecules for cancer therapy, surviving clinical trials and becoming a drug. In general, these filters are based on empirical rules targeting several pharmacokinetic indices that confer crucial information for the speed and success in drug discovery. Herein, we predicted for carvacrol thirteen of the most crucial drug-likeness filters recommended during the design of cancer drug candidates [35, 36], which are shown in Table 4.

Calculated data sets for carvacrol fit well within recommended parameters for an optimal therapeutic option, suggesting the druggability of the carvacrol and demonstrating their potential as likely orally active oncology medicine. According to Lipinski's rule of five (no more than one violation is acceptable) [37], the tested compound could be used as orally dosed drugs in humans. In addition, carvacrol exhibited a great %HIA, which would suggest that the molecule could be absorbed throughout the intestinal segments upon oral administration. This latter statement has been also confirmed by using Caco-2 cell monolayers or MDCK cells as predicting model, in which both models are recommended as a simplified *in vitro* model of intestinal absorption in drug development [38, 39]. In this case, carvacrol displayed recommended values ranges for an ideal drug of 3712 and 2042 nm/s, respectively. Compared to reference values taken from 95% of currently known drugs, carvacrol has an optimal lipophilicity index (LogP_o/w_) of 3.280, may be implying the ability of the molecule to penetrating the lipid bilayers of the malignant cells. This fact was also verified using the calculated polar surface area (PSA) value, which is the most important physicochemical property to correlate passive molecular transport through membranes and drug-membrane interactions [40]. Predicted PSA for carvacrol showed an acceptable therapeutic value of 21.271 Å^2^, indicating again that this compound should have good cellular membrane permeability. Finally, binding to serum albumin (calculated as LogK_HSA_) is the most important indices for distribution and transport of drugs in the systemic circulation. Early prediction of this parameter reduces the amount of wasted time and resources for drug development candidates for anticancer therapy. Notably, carvacrol has a LogK_HSA_ value of 0.023 that fits well within the permitted range for 95% of marketed drugs (from −5 to 2.0). With respect to future pharmaceutical applications, the optimal pharmacokinetic properties make carvacrol a potential therapeutic alternative for specific treatment of breast cancer.

## 3. Materials and Methods

### 3.1. Molecular Modelling Studies

#### 3.1.1. Protein Structure and Setup

To explore the potential mechanism of action of carvacrol, the most representative proteins involved in intracellular signaling pathways driving breast cancer progression, including phosphatidylinositol-3-kinase-*α* wild type (PI3K*α*), human estrogen receptor-*α* (hER-*α*), progesterone receptor (PR), human epidermal growth factor receptor-2 (HER2), the mammalian target of rapamycin (mTOR), and epidermal growth factor receptor (EGFR), were used, respectively. Thus, the crystal structures were obtained from the Protein Data Bank as follows: HER2 (PDB ID: 3PP0) [41], PI3K*α*-wild type (PDB ID: 4JPS) [42], mTOR (PDB ID: 4DRI) [43], hER-*α* (PDB ID: 3ERT) [44], PR (PDB ID: 4OAR) [45], and EGFR (PDB ID: 3POZ) [41]. Discovery Studio (DS) Visualizer 2.5 was used to edit the protein structures and to remove water molecules together with bound ligands. The structures of the selected proteins were parameterized using AutoDockTools [46]. In general, hydrogens were added to polar side chains to facilitate the formation of hydrogen bonds, and the Gasteiger partial charges were calculated.

#### 3.1.2. Ligand Dataset Preparation and Optimization

Ligands used in this study are carvacrol and nine well-known anticancer inhibitors, including six FDA-approved cancer drugs (lapatinib, alpelisib, (−)-rapamycin, 4-OHT, ulipristal acetate, and gefitinib) and two emerging inhibitors for breast cancer therapy: TAK-285 and PIK-93. The selected 2D structures of the ligands were retrieved as CSV files from the PubChem database (https://pubchem.ncbi.nlm.nih.gov/); then DS visualizer was used to rewrite the data files into pdb format. The structures of the ligands were parameterized using AutodockTools to add full hydrogens to the ligands, to assign rotatable bonds, to compute Gasteiger partial atomic charges and save the resulting structure in the required format for use with AutoDock. All possible flexible torsions of the ligand molecules were defined using AUTOTUTORS in AutoDockTools [47] to facilitate the simulated binding with the receptor structure.

#### 3.1.3. Docking and Subsequent Analysis

Docking simulations were performed with AutoDock 5.6 using the Lamarkian genetic algorithm and default procedures for docking a flexible ligand to a rigid protein. Docking calculations were carried out into the binding catalytic site of each protein target. Once potential binding sites were identified, docking of carvacrol to these sites was carried out to determine the most probable and most energetically favorable binding conformations. To accomplish that, rigorous docking simulations involving a grid box to the identified binding site, Autodock Vina 1.1.2 [48], was used. The exhaustiveness was 20 for each protein-compound pair. The active site was surrounded by a docking grid of 32 Å3 with a grid spacing of 0.375 Å. Affinity scores (in kcal·mol) given by AutoDock Vina for carvacrol were obtained and ranked based on the free energy binding theory (more negative value means greater binding affinity). The resulting structures and the binding docking poses were graphically inspected to check the interactions using the DS Visualizer 2.5 (http://3dsbiovia.com/products/) or The PyMOL Molecular Graphics system 2.0 programs.

#### 3.1.4. Molecular Dynamics (MD) Simulation and Free Energy Calculations

In order to verify the molecular interaction stability of mTOR-carvacrol complex, molecular dynamics (MD) simulations were carried out by using the Gromacs program [49] considering the most potential protein target for carvacrol extracted from docking results and the best docking pose. Force field parameters for protein and ligand were derived independently. For the selected protein, the amber03 force field was selected and assigned by using the pdb2gmx tool of the Gromacs program packages; meanwhile, ligand force field parameters were prepared with the generalized AMBER force field (GAFF) using the molecular geometries previously optimized in gas phase using the HF/6-31^∗^ level of theory, [50] with the Gamess-US program [51]. In addition, ligand was verified as a minimum through a harmonic vibrational normal mode analysis. Atomic charges were obtained with the Merz–Kollman scheme [52] by fitting a restricted electrostatic potential (RESP) model [53] by the Gamess-US program, and the output file was used into the resp. subprogram of the AmberTools program package [54]. Assignment of GAFF force field parameters was carried out by the Antechamber program [55] and the required input files for molecular dynamics simulations were prepared using the ACPYPE python interface [56]. Protein and protein-ligand complex were solvated in a rectangular box of TIP3P waters and chloride (Cl−) or sodium (Na+) ions were added to the system by random replacement of water molecules until neutralization of total charge. In order to remove spurious contact, molecular geometries were optimized with the steepest descent algorithm with 100000 steps and protein backbones atoms were constrained with a force constant of 1000 kJ mol^−1^. Then, the MD simulations were allowed to run for 1000 ps in the NpT ensemble. Additionally, 50 ns in the NpT ensemble were calculated for the production stage. All simulations were carried out under periodic boundary conditions. A 12 Å cutoff distance was used to calculate nonbonded interactions. Electrostatic interactions were treated with the Ewald particle mesh (PME) method [57], while van der Waals interactions were introduced by using the cutoff scheme [58]. Finally, the V-rescale thermostat at 300 K with a coupling constant of 1.0 ps was used and the pressure was kept constant at 1 atm using the Parrinello–Rahman barostat [59] with a coupling constant of 2.0 ps and a compressibility factor of 4.5 x 10^−5^ bar-1. All covalent bonds were constrained using the LINCS algorithm and the contact list was updated every 40 fs. The binding free energy was analyzed using the molecular mechanics Poisson−Boltzmann surface area (MM/PBSA) method [60] implemented in Gromacs program. For MMPBSA calculations, the g_mmpbsa software [34] was used for electrostatic interactions, van der Waals interactions, polar solvation energy, and nonpolar solvation energy calculations. The binding free energy was calculated using the last 40 ns of trajectories from the production stage MD simulations, for example, 500 snapshots. The SASA model was used for nonpolar contributions with a surface tension of 0.0226778 (kJ/mol^2^) and a probe radius of 1.4 Å. An ionic strength of 0.150 M·NaCl with radii of 0.95 and 1.81 Å for sodium and chloride ions, respectively, was used for all polar calculations. In addition, dielectric constants of 6, 80 and 1 were used for the protein, water, and vacuum, respectively. To calculate the average binding free energy over the previously selected snapshots, a bootstrap analysis was performed.

#### 3.1.5. Carvacrol Drug Likeness Evaluation

*In silico* drug-likeness indices were evaluated for carvacrol in order to explore its druggability for further clinical studies. To find out the drug-like properties, carvacrol was screened for its pharmacokinetic properties using open-source cheminformatics toolkits such us Molinspiration software (for: MW, rotatable bonds and topographical polar surface area (PSA) descriptors, ALOGPS 2.1 algorithm from the Virtual Computational Chemistry Laboratory (for: Log Po/w descriptor), and Pre-ADMET 2.0 program to predicted various pharmacokinetic parameters and pharmaceutical relevant properties such as apparent predicted intestinal permeability (App. Caco-2), binding to human serum albumin (LogKHSA), MDCK cell permeation coefficients, and intestinal or oral absorption (%HIA). These key parameters define absorption, permeability, motion, and action of drug molecule. The interpretation of two predicted ADMET properties using the Pre-ADMET program was shown in the following.

Value of Caco-2 permeability is classified into three classes:

(1) If permeability < 4, low permeability; (2) if permeability < 70, moderate permeability; and (3) if permeability > 70, higher permeability.

Value of MDCK cell permeability can be classified into three classes:

(1) If permeability < 25, low permeability; (2) if 25 < permeability < 500, moderate permeability; and (3) if permeability > 500, higher permeability.

## 4. Conclusions

Multilevel computational studies suggested that the candidacy of carvacrol for future drugs investigations in the breast cancer treatment should be strongly considered. Virtual screening revealed that mTOR is the main target of carvacrol which has shown a good interaction with this regulating protein with respect to other evaluated proteins responsible of the mammary tumorigenesis. ADME prediction of carvacrol shows that it is a good candidate to oral drug formulation and could be useful as alternative therapy in breast cancer. However, in spite of showing a good prediction on mTOR receptor with values near to rapamycin in docking modeling, our findings in the histological evaluation of our previous research suggest that carvacrol could be protective or preventive against an exposure with any carcinogenic agent. Further studies with other target proteins should be analyzed in order to elucidate how carvacrol is acting in breast cancer.

## Figures and Tables

**Table 1:** Calculated binding affinity for carvacrol and current inhibitors into the active site of the most important targets involved in breast cancer.

	Target	Carvacrol	Lapatinib/TAK-285^*a*^	Alpelisib ^*d*^/PIK-93^*e*^	(−)−Rapamycin (sirolimus)^*c*^	4-OHT ^*f*^	Ulipristal acetate (UPA)	Gefitinib/TAK-285^*a*^
Target protein docking score (kcal/mol)	HER2	−6.6	−10.4/−12	—	—	—	—	—
	PI3K*α*	−6.0	—	−8.1/−7.5	—	—	—	—
	mTOR^*b*^	−7.5	—	—	−8.6	—	—	—
	hER-*α*	−6.3	—	—	—	−9.7	—	—
	PR	−5.4	—	—	—	—	−10.6	—
	EGFR	−6.2	—	—	—	—	—	−8.2/−10.7

^*a*^Potent, selective, ATP-competitive, and orally active HER2 and EGFR inhibitor; ^*b*^mammalian active site of rapamycin was used; ^*c*^specific mTOR inhibitor with IC_50_ of ∼0.1 nM; ^*d*^potent and selective PI3K*α* inhibitor with IC_50_ of 5 nM into the ATP pocket in PI3K*α*; ^*e*^potent PI3K*α* inhibitor (IC_50_ at 19 nM) into the ATP pocket in PI3K*α*; ^*f*^4-hydroxytamoxifen, the active metabolite of tamoxifen.

**Table 2:** Detailed interactions profile between carvacrol and rapamycin at the interface cavity of FKBP12 and mTOR–FRB domain.

	Interactions with FKBP12 protein			Interactions with mTOR–FRB domain		
Ligand	H-bond interactions <3 Å (*n*)	Van der Waals contacts (*n*)	*σ*/*π*-*π*/alkyl interactions (*n*)	H-bond interactions <3 Å (*n*)	Van der Waals contacts (*n*)	*σ/π-π* interactions (*n*)
Carvacrol	0	4Val78, Phe79 Val86, Phe77	0	0	6His2028, Glu2033, Tyr2104, Arg2106, Glu2032, His2106	7Phe2108, Leu2031, Tyr2105, Trp2101, Ser2035
Rapamycin	6Arg73, Tyr113, Ile87, Asp68, Gln85, Gly84	8Val78, Phe79, Leu128, Gly59, Lys121, Phe130, Lys88, Ser118	10Ile122, Phe67, Tyr57, Trp90, Val86, Phe77	0	8His2028, Glu2033, Tyr2104, Leu2097, Gln2099, Trp2101, Tyr2038, Arg2036	4Phe2108, Leu2031, Tyr2105, Phe2039

**Table 3:** Calculated MM/PBSA binding free energy in kcal.mol^−1^ for complex mTOR carvacrol.

Energy contribution	Value (kcal·mol^−1^)
ΔG_vdw_^*a*^	−9.28 ± 1.46
ΔG_Electr_^*b*^	−0.38 ± 0.70
ΔG_Polar_^*c*^	3.85 ± 1.46
ΔG_SASA_^*d*^	−2.21 ± 0.14
ΔG_bind_^*e*^	−18.03 ± 1.57

^*a*^Van der Waals energy terms. ^*b*^Electrostatic energy contribution. ^*c*^Polar contributions between the solute and solvent to the solvation energy. ^*d*^Nonpolar solvation energy using the solvent accessible surface area. ^*e*^ΔG_bind_ is the total free binding energy.

**Table 4:** Drug-likeness evaluation of carvacrol.

Properties	Carvacrol
MW^*a*^	150.220
PSA^*b*^ (7−200 Å2)	21.271
*n*−Rot bond (<10)	2
*n*−ON^*c*^ (<10)	1
*n*−OHNH^*d*^ (<5)	1
Log po/w ^*e*^ (−2.0–6.5)	3.280
LogKHSA ^*g*^ (−1.5−2.0)	0.023
Caco−2 ^*h*^ (nm/s) <25 poor; >500 great	3712
App. MDCK (nm/s)^*I*^ <25 poor, >500 great	2042
% HIA^*j*^	100
Lipinski's rule of five (≤1)	0
% HOA ^*k*^ >80% is high <25% is low	>80

^*a*^Molecular weight of the molecule; ^*b*^polar surface area; ^*c*^*n*-ON number of hydrogen bond acceptors; ^*d*^*n*-OHNH number of hydrogen bonds donors; ^*e*^predicted octanol-water partition coefficient; ^*f*^aqueous solubility; ^*g*^in vitro binding constant to human serum albumin; ^*h*^predicted human intestinal permeability model (nonactive gut-blood barrier transport; ^*i*^apparent permeability across cellular membranes of Madin–Darby Canine Kidney (MDCK) cells; ^*j*^ human intestinal absorption (% HIA); ^*k*^ percent of human oral absorption (HOA %).

## Data Availability

The data that support the findings of this study are available from the corresponding author upon reasonable request.

## Supplementary Materials

Supplementary Materials S1: 2D-binding interactions maps from MD simulations for carvacrol-mTOR complex. S2: 3D plots for comparison pose docking, snapshot of carvacrol backbone atoms at 10 and 20 ns, and after 50 ns MD simulations for carvacrol-mTOR complex. Best docking pose (red) snapshot at 10 ns (grey), snapshot at 20 ns (orange), and pose post-50 ns MD simulation (yellow). S3: 3D plots for comparison pose docking and pose after MD simulations for carvacrol-mTOR complex (best docking pose in red and pose post-50 ns MD simulation in yellow). S4: the individual energy contributions of each residue from MM/PBSA calculations in carvacrol-mTOR complex. FKBP12 protein is colored blue and FRB domain green.
