# Supplementary figures and images for: Carvacrol: An In Silico Approach of a Candidate Drug on HER2, PI3Kα, mTOR, hER-α, PR, and EGFR Receptors in the Breast Cancer
Source: Evid Based Complement Alternat Med. 2020 Oct 26;2020:8830665. doi: 10.1155/2020/8830665 (PMC7607278; doi:10.1155/2020/8830665)

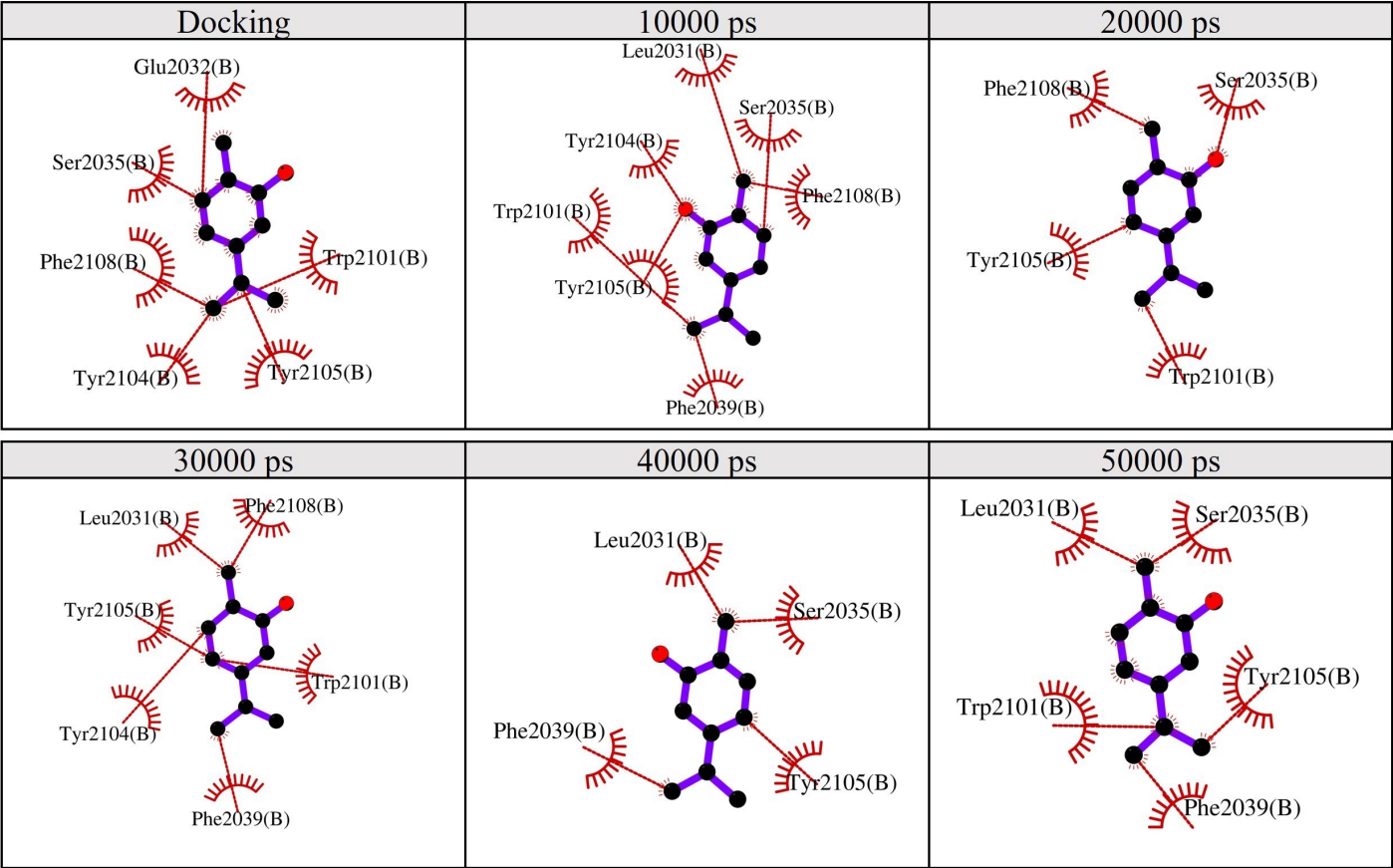

Supplement: Supplementary Materials — S1: 2D-binding interactions maps from MD simulations for carvacrol-mTOR complex. S2: 3D plots for comparison pose docking, snapshot of carvacrol backbone atoms at 10 and 20 ns, and after 50 ns MD simulations for carvacrol-mTOR complex. Best docking pose (red) snapshot at 10 ns (grey), snapshot at 20 ns (orange), and pose post-50 ns MD simulation (yellow). S3: 3D plots for comparison pose docking and pose after MD simulations for carvacrol-mTOR complex (best docking pose in red and pose post-50 ns MD simulation in yellow). S4: the individual energy contributions of each residue from MM/PBSA calculations in carvacrol-mTOR complex. FKBP12 protein is colored blue and FRB domain green. [file 8830665.f1.zip › 8830665.f1/Supplementary material Figure 1.pdf]

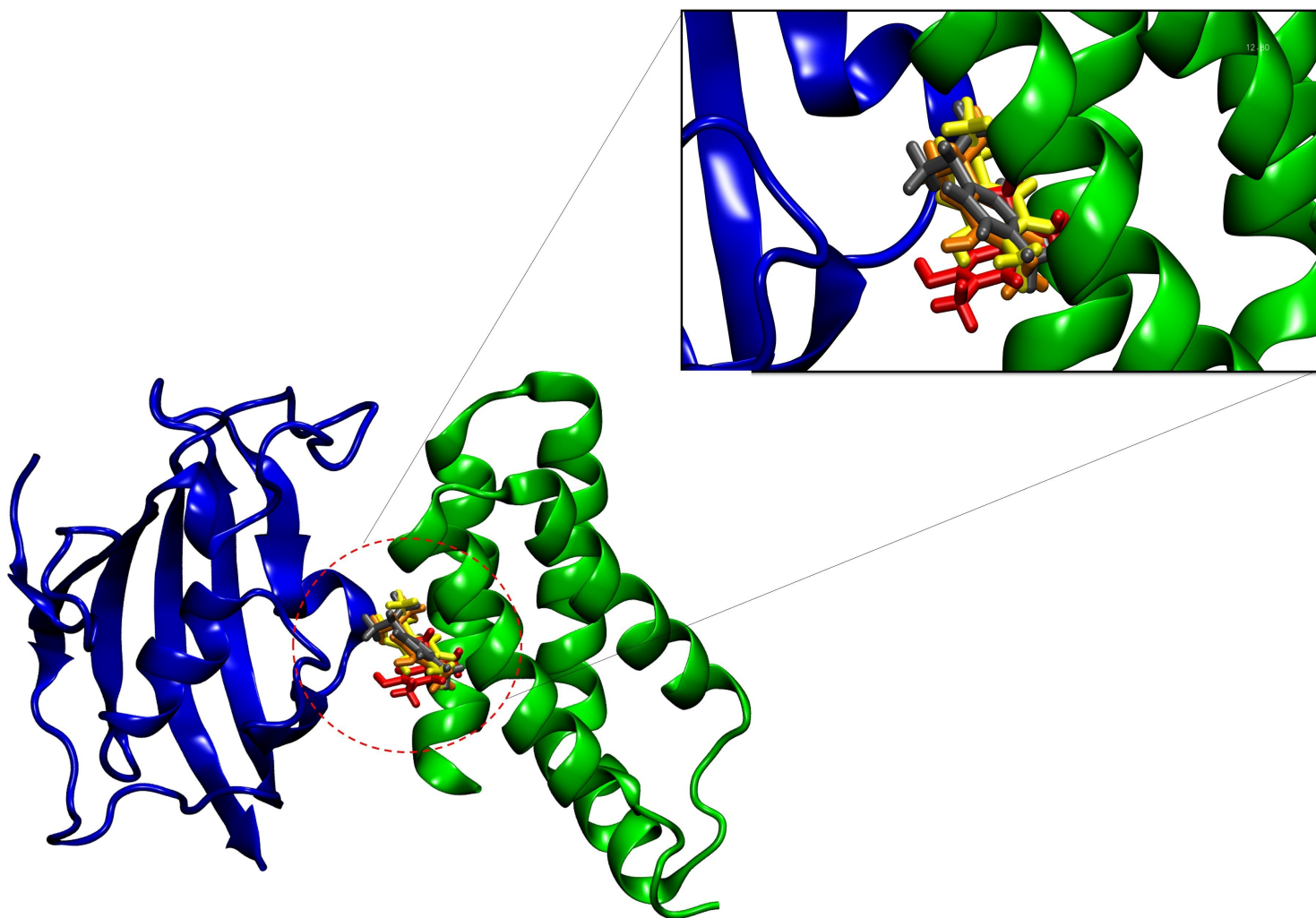

Supplement: Supplementary Materials — S1: 2D-binding interactions maps from MD simulations for carvacrol-mTOR complex. S2: 3D plots for comparison pose docking, snapshot of carvacrol backbone atoms at 10 and 20 ns, and after 50 ns MD simulations for carvacrol-mTOR complex. Best docking pose (red) snapshot at 10 ns (grey), snapshot at 20 ns (orange), and pose post-50 ns MD simulation (yellow). S3: 3D plots for comparison pose docking and pose after MD simulations for carvacrol-mTOR complex (best docking pose in red and pose post-50 ns MD simulation in yellow). S4: the individual energy contributions of each residue from MM/PBSA calculations in carvacrol-mTOR complex. FKBP12 protein is colored blue and FRB domain green. [file 8830665.f1.zip › 8830665.f1/Supplementary material Figure 2.pdf]

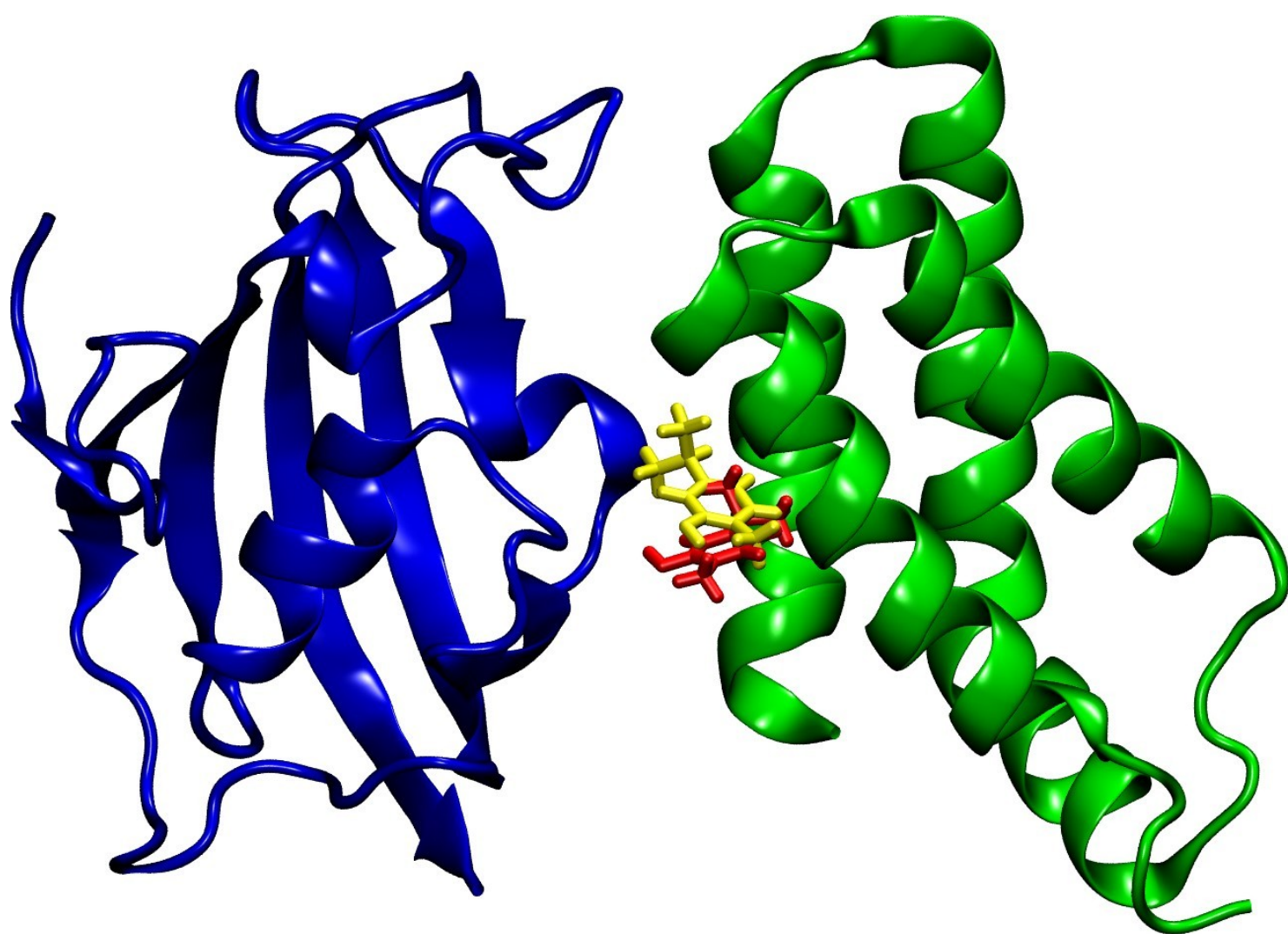

Supplement: Supplementary Materials — S1: 2D-binding interactions maps from MD simulations for carvacrol-mTOR complex. S2: 3D plots for comparison pose docking, snapshot of carvacrol backbone atoms at 10 and 20 ns, and after 50 ns MD simulations for carvacrol-mTOR complex. Best docking pose (red) snapshot at 10 ns (grey), snapshot at 20 ns (orange), and pose post-50 ns MD simulation (yellow). S3: 3D plots for comparison pose docking and pose after MD simulations for carvacrol-mTOR complex (best docking pose in red and pose post-50 ns MD simulation in yellow). S4: the individual energy contributions of each residue from MM/PBSA calculations in carvacrol-mTOR complex. FKBP12 protein is colored blue and FRB domain green. [file 8830665.f1.zip › 8830665.f1/Supplementary material Figure 3.pdf]

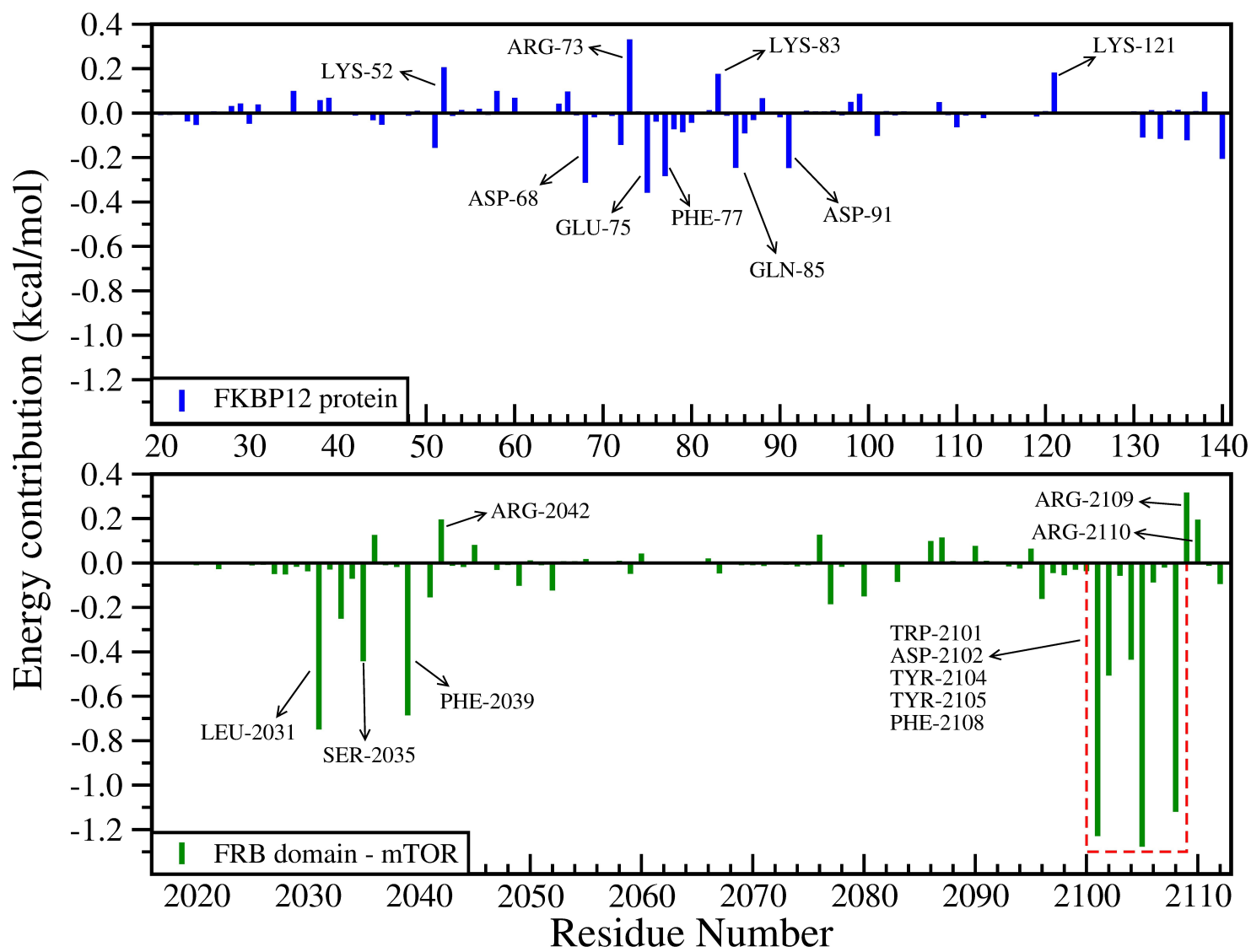

Supplement: Supplementary Materials — S1: 2D-binding interactions maps from MD simulations for carvacrol-mTOR complex. S2: 3D plots for comparison pose docking, snapshot of carvacrol backbone atoms at 10 and 20 ns, and after 50 ns MD simulations for carvacrol-mTOR complex. Best docking pose (red) snapshot at 10 ns (grey), snapshot at 20 ns (orange), and pose post-50 ns MD simulation (yellow). S3: 3D plots for comparison pose docking and pose after MD simulations for carvacrol-mTOR complex (best docking pose in red and pose post-50 ns MD simulation in yellow). S4: the individual energy contributions of each residue from MM/PBSA calculations in carvacrol-mTOR complex. FKBP12 protein is colored blue and FRB domain green. [file 8830665.f1.zip › 8830665.f1/Supplementary material Figure 4.pdf]
